# P01.28. The effect of 650nm and 10.6μm CO_2_ laser irradiation on knee osteoarthritis C57 black mouse in serum prostaglandin E2 and hyaluronic acid

**DOI:** 10.1186/1472-6882-12-S1-P28

**Published:** 2012-06-12

**Authors:** X Shen

**Affiliations:** 1 Shanghai University of Traditional Chinese Medicine, Shanghai, China

## Purpose

To observe the effects of two different wavelength lasers exertion on Hyaluronic Acid (HA) and Prostaglandin E2 (PGE2) in serum when they were irradiated on Dubi (ST35) of C57 black mouse with knee osteoarthritis (KOA).

## Methods

60 healthy C57 black mice were randomly allocated to 6 groups including control group, model group, 10.6μm CO_2_ laser group, 650nm laser group, combined laser group (combined 10.6μm CO_2_ laser and 650nm laser) and sham group. Each treating group received irradiation from different lasers at Dubi (ST35) for 90s in a total of 15 sessions. The sham group also received treatment with the laser probe putting at Dubi (ST35) for 90s in a total of 15 sessions.

## Results

Compared with the model group, the PGE2 in serum of the following three groups all significantly decreased (p<0.05): 10.6μm CO2 laser group, 650nm laser group and the combined laser group. The same significant decrease (p<0.05) was found in the sham laser group. No significant differences of serum HA were found between the model group and control group. But compared with the model group, the serum HA significantly increased(p<0.05).

## Conclusion

There is an obvious effect of 10.6μm CO_2_ laser, 650nm laser and combined laser group in reducing the serum PGE2. Yet the three lasers have no obvious effect on serum HA. However, the relation of the efficacy of those three lasers and the HA content in joint and serum is to be tested in the future.

